# HIV Case Notification Rates in the Kingdom of Saudi Arabia over the Past Decade (2000–2009)

**DOI:** 10.1371/journal.pone.0045919

**Published:** 2012-09-26

**Authors:** Mohammed A. A. l. Mazroa, Ibrahim A. Kabbash, Sanaa M. Felemban, Gwen M. Stephens, Raafat F. Al-Hakeem, Alimuddin I. Zumla, Ziad A. Memish

**Affiliations:** 1 Field Epidemiology Training Program, Saudi Arabia Ministry of Health, Riyadh, Kingdom of Saudi Arabia; 2 Faculty of Medicine, Tanta University, Tanta, Egypt; 3 Public Health Directorate, Ministry of Health, Saudi Arabia Ministry of Health, Riyadh, Saudi Arabia; 4 University of British Columbia, Vancouver, Canada; 5 Department of Infection, University College London, London, United Kingdom; University of Modena & Reggio Emilia, Italy

## Abstract

**Objective:**

To study trends in HIV case notification rates in the Kingdom of Saudi Arabia.

**Design:**

A ten year retrospective review of annual HIV case notification returns to the Ministry of Health, Kingdom of Saudi Arabia.

**Methods:**

Annual Registry statistics covering the period 2000 to 2009 were reviewed. Annual incidence trends were stratified according to the following demographics: age, nationality, geographical region of residence, gender, and mode of disease acquisition.

**Results:**

10,217 new HIV cases (2,956 in Saudi nationals and 7,261 in non-Saudis) were reported. Africans of Sub-Saharan Africa origin accounting for 3,982/7,261 (53%) of non-Saudi cases constituted: Ethiopians (2,271), Nigerians (1,048), and Sudanese nationals (663). The overall average annual incidence was <4 cases per 100,000; 1.5 cases per 100,000 for Saudis (range 0.5–2.5), and 13.2 per 100,000 for non-Saudis (range 5.7–19.0). Notifications increased yearly from 2000 for both groups until a plateau was reached in 2006 at 1,390 new cases. Case notification in Saudi nationals increased from 20% in the early 2001 to 40% in 2009. 4% (124/2,956) of cases were reported in Saudi children. The male to female ratio was 1.6∶1 for non-Saudi nationals (43.8% male, 27.3% female) and 4.4∶1 for Saudis (23.5% male, 5.4% female).

**Conclusions:**

Whilst the numbers of reported HIV cases have stabilised since 2006, HIV/AIDS remains an important public health problem in KSA, both in migrants and Saudi nationals. HIV transmission to Saudi children is also of concern. Optimization of data collection, surveillance, and pro-active screening for HIV is required.

## Introduction

The aims of the World Health Organisation (WHO) and UNAIDS global health sector strategy on HIV/AIDS, 2011–2015 [1], [2] are to achieve universal access to HIV prevention, diagnosis, treatment and care interventions for all in need, and to contribute to achieving health-related Millennium Development Goals and their associated targets by 2015. The WHO strategic direction [1] is composed of core elements to: a) Revolutionize HIV prevention, b) Eliminate new HIV infections in children, c) Catalyse the next phase of treatment, care and support, d) Provide comprehensive and integrated services for key populations. The WHO strategy emphasises that given the widely differing characteristics of the epidemics between countries and regions, national responses must be guided by the most current strategic information on the nature of the HIV epidemic and the country context. “Knowing the epidemic” thus includes understanding where, how and among whom new infections are occurring. It also requires identifying the social, legal and economic conditions that increase the risk of HIV transmission and limit access to HIV information and services. National responses must take into consideration: the preparedness, infrastructure and capacity of the health system or health systems; whether the current response meets the needs of those most vulnerable to and at risk of HIV infection”.

Accurate characterisation of the epidemiological features of the HIV epidemic in the Middle East has been slow due to social, cultural, taboo and religious factors [3]. The Kingdom of Saudi Arabia (KSA) occupies more than 80% of the Arabian Peninsula and covers an area of 2.25 million square kilometres. The population of Saudi Arabia is estimated to be around 25.7 million, of which 5.5 million (∼20%) are non-Saudi expatriate workforce residents recruited from diverse geographical backgrounds. For the period 1984 through 2001, a total of 6046 cases with HIV infection were reported from the KSA: 1285 (21.3%) cases among Saudi citizens and 4761 (78.7%) cases among expatriates [4]. Since 1984 HIV screening was made routine for certain groups of individuals, including suspected patients with HIV/AIDS, risk-related contacts of HIV-infected persons, blood donors, staff in certain occupations, new migrants, and intravenous drug users. In the late 1990s the KSA government started increasing its public education and prevention campaign. Pre-marital and pre-natal HIV testing programs were established. Notification of HIV/AIDS cases in KSA was made mandatory by the government since 1984 and confirmed HIV-positive cases are through regional health authorities to the Ministry of Health National HIV/AIDS Program.

In 2007, the Regional WHO Eastern Mediterranean Region Office focused its efforts on providing guidance according to specific country needs for expanding access to HIV care and treatment, testing and counseling and prevention of HIV transmission [5]. The Regional Office collects quarterly HIV and AIDS case reports from countries in the Region. At end of 2007, the estimated number people living with HIV (PLHIV) in the Eastern Mediterranean Region reached 530 000 (420 000–700 000), and an estimated 55 000 (28 000–110 000) new HIV infections occurred in 2007 [4]. Most countries of the region have weak data collecting and surveillance systems, and the data remain estimates only with wide margins.

In the past 5 years the KSA government has provided a more proactive programmatic response and demonstrated increased level of commitment and support [6]. To evaluate current trends in HIV notification rates, and their stratification by age, nationality, geographical region of residence, gender, and mode of disease acquisition, we undertook a ten year retrospective review of annual HIV case notification returns to the Ministry of Health, Kingdom of Saudi Arabia.

## Methods

A ten year retrospective review of annual HIV case notification returns to the National AIDS Program, Ministry of Health, Kingdom of Saudi Arabia. Annual Registry statistics covering the period 2000 to 2009 were reviewed. Annual incidence trends were stratified according to the following demographics: age, nationality, geographical region of residence, and gender. Notification of HIV/AIDS cases was made mandatory by the KSA government since 1984. Confirmed positive cases are reported to regional health authorities and through regional AIDS coordinators to the National AIDS Program.

## Results

### Ethics statement

No ethics was requires since this was an unlinked, anonymised case note and database review which did not involve patients. The Ministry of Health Ethcis Committee had adavised us as such. Data collected were unlinked and anonymised.

### New HIV cases in KSA

**Figure 1:**
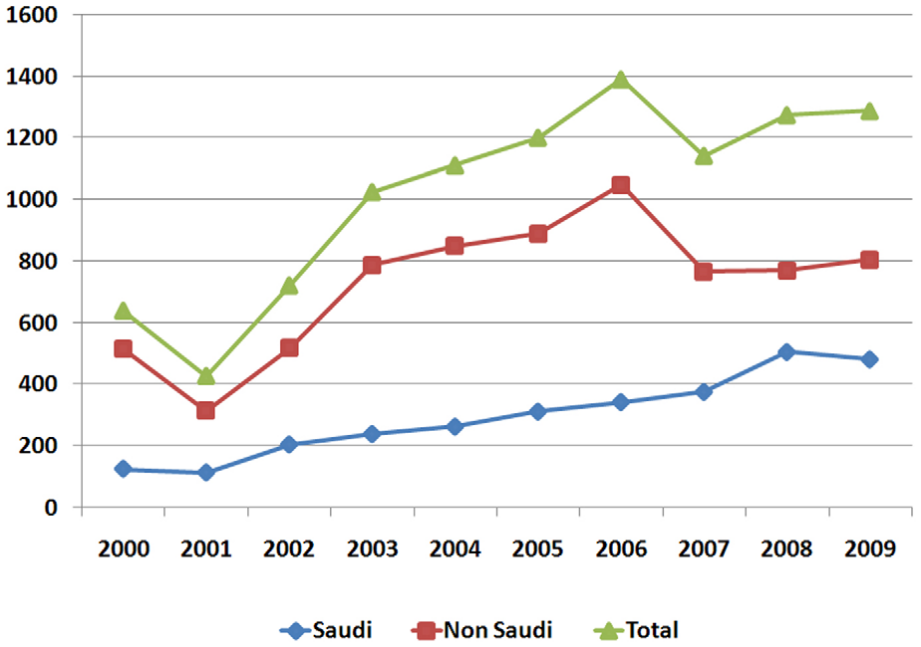
Yearly HIV Case notifications for Saudi Arabia (2000–2009).

A total of 10,217 new HIV cases were reported for years 2000 through 2009. Yearly notifications by gender and national origin are shown in **table 1**. The overall annual incidence for the decade was 4 cases per 100,000. **Figure 1** depicts trends in HIV case notification rates from 2000 to 2009.

**Table 1:** Yearly HIV case notification rates by nationality and gender.

	Non-Saudi			Saudi			All
Year	Total	Female	Male	Total	Female	Male	Total
**2000**	514	214	300	125	20	105	**639**
**2001**	314	137	177	113	30	83	**427**
**2002**	517	160	357	204	32	172	**721**
**2003**	787	298	489	238	37	201	**1,025**
**2004**	849	367	482	262	59	203	**1,111**
**2005**	890	367	523	311	81	230	**1,201**
**2006**	1,048	331	717	342	56	286	**1,390**
**2007**	767	238	529	375	64	311	**1,142**
**2008**	769	242	526	505	91	414	**1,274**
**2009**	806	323	483	481	94	387	**1,287**
**TOTAL**	**7,261**	**2,677**	**4,583**	**2,956**	**564**	**2,392**	**10,217**

### Saudi versus non-Saudi HIV cases

The number of reported HIV cases in KSA during the ten year period indicates more cases among non-Saudis than among Saudis. There were 2,956 infections in Saudi nationals and 7,261 in non-Saudis giving a rate of 1.5 cases per 100,000 for Saudis (range 0.5 to 2.5), and 13.2 per 100,000 for non-Saudi residents (range 5.7 to 19.0).

### HIV Trends over 10 years

Case reports increased each year for both groups until a statistical plateau was reached in 2006 with 1,390 new cases [**Figure 1**]. However, the pattern over the past ten years is changing, with a declining number of new cases among non-Saudis over time, and an increasing number of cases among Saudis (although there is a downward dip among both Saudis and non-Saudis after 2009). The contribution of Saudis to total positives increased from 20% in the early years of the decade to 40% in 2008 and 2009.

### HIV by Gender

Males accounted for two thirds of all new cases during the 2000–2009 decade, with non-Saudi males accounting for the majority of new diagnoses (**Table 1**). The male to female ratio was 1.6∶1 for non-Saudi nationals (43.8% male, 27.3% female) and 4.4∶1 for Saudis (23.5% male, 5.4% female).

### Non-Saudi cases

Africans of sub-Saharan origin accounted for 53% of 7,261 non-Saudi HIV cases, of which the majority were Ethiopians (2,271), Nigerians (1,048), and Sudanese nationals (663). Twenty two percent of cases were in South Asians: India (634), Bangladesh (512) and Pakistan (462). Fifteen percent of new cases were reported in Yemeni nationals (1048), and the remaining 10% were Indonesians (357) and ‘other nationalities constituted 375.

### Saudi cases

#### Age distribution

A total of 2,355 out of 2956 (80%) of Saudi cases were in adults between the ages of 15 and 49 years. There were 124 (4%) cases of HIV reported in Saudi children. Saudi adults aged 50 years or older accounted for 16% (477) of cases. A large percentage of cases (70%) were diagnosed prior to onset of advanced infection or AIDS diagnosis from screening programs.

#### Saudi cases according to geographical region

Regional differences were pronounced with the two major cities Jeddah and Riyadh reporting more than half (56%) of all new cases, 1,395 (47%) and 549 (9%) respectively. Lower numbers were reported from the Western region: Jeddah-Makkah (192), Taif (112) and Medina (60). Southern districts of Jazan and Aseer reported 136 and 125 new cases respectively (8.8%) of total cases. The Eastern region, including smaller cities of Dammam, Daharan, Al Khobar, had a total of 201 new cases (6.8%) of total cases.

#### Risk factors for Saudi nationals

Information on behavioral risk factors was poorly documented and could only be identified from registry records in 46%. Sexual transmission was reported for 40%, and 6% cited injection drug use as a risk factor. One hundred twenty-four new infant cases (4% of the total) were documented as a result of mother to child transmission.

## Discussion

The main findings of our study are that: a) over a 10 year period (2000 to 2009) compared to sub-Saharan Africa and Europe, KSA remains a low HIV prevalence country with an average annual incidence of <4/100,000; b) low HIV incidence is seen among both Saudi nationals and non-Saudis residents; c) HIV infection of the non-Saudi residents appears to mirror generalized HIV epidemics in their countries of origin, with highest disease rates in migrants from sub Saharan Africa. Gender ratios for migrants also mirror those of countries with generalized transmission where female and male cases are roughly equal in number; d) whilst non-Saudi nationals account for a large percentage of the HIV cases, the Saudi contribution to total cases has increased from 20% in early years of the decade to 40% in 2009, e) the male- to female ratio in Saudi nationals has increased from 3∶1 in 2003 to 4.4∶1 in 2009; and f) Despite available prenatal screening and routine antiviral prophylaxis of seropositive mothers, mother to child transmission still occurs with 124 notified cases of HIV in Saudi children. No specific data were available on mode of transmission.

There were several limitations of this study which might have led to underestimation of the true number of HIV cases. The ability to estimate accurately the number of people living with HIV in KSA remains a challenge. The data needs to be interpreted in light of the data source, and the number of Saudis and non-Saudis who are tested each year. The KSA HIV/AIDS program relies mainly on reported HIV case data to track the epidemic. These data reflect a combination of people seeking medical treatment for HIV-related conditions or suspected HIV, and HIV infections that are found through routine testing. HIV-infected cases are passively detected in KSA by routine testing certain groups of individuals, including risk-related contacts of seropositive persons, blood donors, prisoners, staff in certain occupations, newcomers to the country and substance abusers in rehabilitation centres [7]–[12]. As in other Middle Eastern countries, cultural and religious reasons and the social barriers, stigmatization and discrimination associated with HIV, prevent individuals from seeking voluntary HIV testing and counseling. Non-Saudis are tested systematically when they migrate to KSA legally for work. However, the problem of HIV in illegal migrants remains a problem since most of them avoid the authorities and do not access care. Non-Saudi migrant workers who acquire HIV infection after taking up employment are detected on re-testing when the person renews their residence status. Others are detected through routine testing. Other vulnerable subsets of the population such as homosexuals and intravenous drug abusers may be reluctant to access health care. As a result, national figures obtained through surveillance records may be inaccurate, and under-reporting is likely.

There are several possible explanations for the narrowing of the gap over the period 2004–2009 between the number of Saudi and non-Saudi HIV cases. This increase in Saudis may be due to the fact that more HIV testing is being done, or it could be the result of increasing numbers of infections, or a combination of the two. The number of Saudis being tested has increased as greater awareness and testing opportunities have been created through provider initiated voluntary testing in health facilities and greater uptake of pre-marital testing facilities in KSA. Therefore, the increased infections among Saudis could be merely the result of more acceptability and voluntary seeking of testing among Saudis (relative to non-Saudis). The data seem to suggest that the incidence of HIV among Saudis is not steadily stable, but rather may be rising over time. It will be important to monitor patterns over the next few years will be to monitor numbers and profiles of people being tested over time.

A factor that provides some insight into HIV transmission dynamics in a country is the ratio of HIV infected males vs. females. Non-Saudi male to female gender ratio was 1.3∶1 whilst the ratio of HIV-infected males to females among non-Saudis is lower than that of Saudis, and has also remained constant over the past ten years (around 4∶1), implying that there are more males than females infected. Potential statistical biases include the fact that males are more likely to be tested, i.e. in the course of employment, during military service or as blood donors. It is anticipated that the male to female ratio will decrease, as more infected males infect their female sexual partners. However, national reporting does not currently identify the number of women diagnosed following a husband's diagnosis. Travel outside KSA by Saudi males is considered to be a risk factor for transmission of HIV infection to female partners and the issue of HIV screening for this group is a vexed one. More testing opportunities are needed to identify discordant couples, particularly investigations of male partners to reduce disease risk in women of childbearing age.

The rate of mother-to-child transmission of human immunodeficiency virus (HIV) type 1 has been reported to be high in Saudi Arabia [8]. Our data show 124 pediatric (infant) Saudi cases between 2000 and 2009, or an average annual incidence of 0.5/100,000. A voluntary HIV testing program for pregnant women has been available since 2006; antiviral prophylaxis is available at no cost for seropositive cases. Despite this, a dozen HIV positive children are identified each year, each one representing two transmission events. This suggests there are significant prevention gaps, i.e. missed screening opportunities, non-compliance with antiviral prophylaxis, treatment failure, or newly acquired maternal infection during pregnancy. Implementing a universal prenatal screening program, particularly one that includes testing for both parents would address the latter problem. Eliminating maternal and pediatric HIV infection is goals well within Saudi Arabia's reach [8]. Comprehensive approaches to preventing mother-to-child transmission of HIV [1], [2], including setting national targets to eliminate HIV in children using national prevention and treatment protocols are being developed. Key components include preventing HIV infection in women of child-bearing age, preventing unintended pregnancies among women living with HIV, reducing HIV transmission from women living with HIV to their infants, and providing appropriate early treatment and care for women living with HIV, their children and families [1], [2].

Due to the unique socio-cultural and religious context in KSA and non-acceptance of individuals with HIV-related risk behaviors, HIV proper prevention programs remains a challenge. However, there are still active prevention responses with many highlights and progress over the past two years including: 1) greatly increased involvement of civil society and NGOs, 2) strong efforts to integrate HIV services into health facilities, and 3) engagement of the National AIDS Program with multi-sectoral partners. During the past 10 years the activities of the National AIDS Program has been significantly scaled up and now constitutes a strong, well-managed central unit with more staff, resources and better capacity [6]. This, combined with increased political support, have resulted in more visibility for the program, more involvement of civil society, and ultimately more open discussion of HIV. Prevention programs, run by the NGOs started in Jeddah and Riyadh are expanding through satellite units and branches in other cities. The National AIDS Program has engaged proactively with multi-sectoral partners including media, faith-based organizations, the Ministry of Interior, Ministry of Social Affairs and the Ministry of Sports and Youth Affairs. In addition to integration of Voluntary Testing and Counseling (VCT), there is widespread scale-up of separate VCT services.

### Conclusions

Health authorities acknowledge that effective prevention programs are a complicated due to social barriers, stigmatization and discrimination associated with the disease, all of which may prevent individuals from seeking voluntary HIV testing and counselling. By law, every Saudi citizen who is infected with HIV or has AIDS is entitled to free medical care and has protection of their privacy as to how they got infected. The Kingdom of Saudi Arabia continues to mobilize resources towards HIV/AIDS education and prevention measures and for improved HIV/AIDS services in an effort to meet Millennium Development Goals including a 2015 target to halt the spread of HIV. Our retrospective data extracted by our study from National annual returns has inherent biases but provides useful information from which the National AIDS Program is being advanced. The effectiveness of any HIV/AIDS program can only be assessed by having accurate evidence base of continued transmission of HIV within the community. There is a need for generating more accurate and comprehensive evidence-based information which will be essential for guiding rational planning and resource allocation.
